# Shengmai Yin Attenuates Hypobaric Hypoxia-Induced Erythrocyte Echinocytosis by Restoring Redox Homeostasis and Normalizing Band 3–Syk/SHP2 Interactions

**DOI:** 10.3390/antiox15091205

**Published:** 2026-09-20

**Authors:** Jie Ding, Jiali Hua, Lingyi Meng, Chenyan Lu, Jiawei Liu, Zenghua Lin, Guohua Wang, Qianqian Luo

**Affiliations:** 1 Department of Hypoxic Biomedicine, Institute of Special Environmental Medicine, Nantong University, Nantong 226001, China; 2225310004@stmail.ntu.edu.cn (J.D.); hjl-hys@stmail.ntu.edu.cn (J.H.); mly9947@stmail.ntu.edu.cn (L.M.); elijahwu@stmail.ntu.edu.cn (C.L.); ljw@stmail.ntu.edu.cn (J.L.); 2 Department of Hematology, Affiliated Hospital of Nantong University, Nantong University, Nantong 226001, China; linzenghua@ntu.edu.cn; 3 National Stroke Administration Office, Xuanwu Hospital Capital Medical University, Beijing 100053, China

**Keywords:** Shengmai Yin, hypobaric hypoxia, erythrocyte, echinocytosis, oxidative stress, superoxide, Band 3, oxygen transport

## Abstract

Acute exposure to high-altitude hypobaric hypoxia (HH) disrupts redox homeostasis and impairs erythrocyte morphology and oxygen transport. Shengmai Yin (SMY) is a botanical formulation with reported antioxidant activity, but its effects on HH-induced erythrocyte injury remain unclear. Male C57BL/6J mice received SMY in drinking water for 3 days before 24 h of simulated HH equivalent to 6000 m. Complementary in vivo and in vitro experiments assessed redox indices, lipid peroxidation, hemoglobin redox status, erythrocyte morphology, arterial oxygenation, and Band 3-associated protein interactions. HH increased serum DHE-derived fluorescence, nitric oxide, and malondialdehyde, reduced antioxidant capacity, induced echinocytosis, decreased oxyhemoglobin, and increased methemoglobin. Among the tested redox challenges, 2-phenyl-4,4,5,5-tetramethylimidazoline-1-oxyl 3-oxide (PTIO) most closely reproduced the combined morphological and hemoglobin abnormalities. SMY scavenged DPPH, hydroxyl-radical, and superoxide-related signals in cell-free assays; reduced PTIO- and hypoxia-induced erythrocyte damage; and reversed established echinocytosis within 30 min in vitro. In mice, SMY reduced echinocytosis and improved arterial oxygenation without detectable short-term hepatic, renal, or histological toxicity. SMY also normalized HH- and PTIO-induced changes in Band 3 interactions with Syk and SHP2. These findings support SMY as a redox-modulating intervention that preserves erythrocyte structure and function during acute HH.

## 1. Introduction

Redox homeostasis reflects the balance between reactive oxygen species (ROS) generation and antioxidant defenses. When ROS production exceeds cellular or systemic antioxidant capacity, oxidative modification of lipids, proteins, and nucleic acids can impair tissue function and promote inflammatory injury [1,2,3,4]. At high altitude (HA), reduced barometric pressure lowers inspired and arterial oxygen tension, producing hypobaric hypoxia (HH) and favoring ROS generation [5,6,7]. Acute HA exposure is associated with increased lipid peroxidation, reduced antioxidant enzyme activity, and symptoms of acute mountain sickness [8]. In healthy trekkers, HA exposure has also been associated with increased ROS and nitric oxide-derived metabolites, consistent with disruption of redox homeostasis [9].

Erythrocytes are the principal carriers of oxygen and require a flexible biconcave shape for efficient gas exchange and microvascular transit [10,11,12]. Oxidative injury can alter membrane lipids and proteins, reduce deformability, impair oxygen delivery, and accelerate erythrocyte aging [13,14]. Erythrocyte-derived redox signals may also amplify inflammatory responses in adjacent tissues [15]. Band 3, the major erythrocyte anion exchanger, links the membrane to the spectrin-actin cytoskeleton through ankyrin and participates in oxygen-dependent organization of glycolytic enzymes [16]. Hypobaric hypoxia and reoxygenation can impair Band 3 function and reduce its association with glyceraldehyde-3-phosphate dehydrogenase (GAPDH) [17]. HA exposure has additionally been associated with changes in erythrocyte size heterogeneity, viscosity, filtration time, and lipid peroxidation [18,19,20]. However, the redox species and membrane-protein interactions that contribute to acute HH-induced echinocytosis remain incompletely defined. Band 3 also functions as an oxygen- and redox-responsive membrane signaling hub. Prior studies link Band 3 tyrosine phosphorylation to changes in membrane-cytoskeletal organization, metabolic enzyme binding, and broader erythrocyte phosphoproteome remodeling [21,22,23]. Oxygenation state can regulate Band 3-associated interactions, whereas oxidant exposure can promote hemoglobin oxidation together with Band 3 tyrosine phosphorylation [24,25]. These observations provide the rationale for examining the association of Band 3 with the kinase Syk and the phosphatase SHP2 in the present HH model.

To model distinct redox challenges, we compared 2-phenyl-4,4,5,5-tetramethylimidazoline-1-oxyl 3-oxide (PTIO), hydrogen peroxide (H_2_O_2_), and 2,2-diphenyl-1-picrylhydrazyl (DPPH). PTIO is a redox-active nitroxide commonly used as a nitric oxide scavenger and can perturb the balance between nitric oxide and superoxide-related pathways [26]. H_2_O_2_ provides a peroxide challenge and can generate hydroxyl radicals in the presence of transition metals, whereas DPPH is a stable nitrogen-centered radical. Comparing these agents allowed us to identify an operational in vitro condition that reproduced the combined morphological and hemoglobin abnormalities observed during HH. Oxidant-challenge/antioxidant-rescue paradigms are well established in erythrocyte research; H_2_O_2_-based models, for example, have been used to test antioxidant preservation of Band 3 function and hemoglobin redox status, and recent work has summarized redox-targeted strategies for maintaining RBC integrity [27,28].

Shengmai Yin (SMY; also known as Shengmai San) is a traditional botanical formulation composed of *Panax ginseng*, *Ophiopogon japonicus*, and *Schisandra chinensis*. Botanical products with antioxidant activity have shown protective effects in diverse experimental settings [29,30,31,32,33,34,35], and ginseng total saponins can improve oxidant-induced erythrocyte deformability [36]. SMY contains multiple redox-active constituents, including ginsenosides and lignans, together with mineral elements that may influence redox reactions [37,38,39,40]. Nevertheless, its effects on HH-induced erythrocyte injury and the associated membrane-protein interactions have not been established.

We hypothesized that SMY would attenuate acute HH-induced erythrocyte injury by limiting oxidative stress and preserving Band 3-associated membrane organization. We therefore evaluated its effects on erythrocyte morphology, lipid peroxidation, hemoglobin redox status, arterial oxygenation, and the interactions of Band 3 with spleen tyrosine kinase (Syk) and Src homology 2 domain-containing phosphatase 2 (SHP2) in complementary in vivo and in vitro models.

## 2. Materials and Methods

### 2.1. Animals, SMY Administration, and Hypobaric Hypoxia Exposure

A total of 150 eight-week-old male C57BL/6J mice (weighing approximately 25 g) were obtained from the Animal Experimental Center of Nantong University. Animals were randomly assigned to normobaric normoxia (NN; ambient pressure and approximately 21% O_2_) or hypobaric hypoxia (HH) groups after body-weight balancing. HH was produced in a decompression chamber (ProOx-810, Tow-Int Technology Co., Ltd., Beijing, China) at conditions equivalent to an altitude of 6000 m for 24 h [41,42]. The ascent rate was 5 m/s, and the system reached the simulated altitude of 6000 m within approximately 20 min. Chamber ventilation was maintained at 23 air exchanges per minute. NN mice were maintained under otherwise comparable conditions without decompression. SMY (Tongrentang, Beijing, China; National Drug Approval No. Z11020363) was diluted 1:30 in drinking water and provided ad libitum beginning 3 days before HH exposure and throughout the exposure period. The SMY drinking solution was prepared fresh daily. In pilot observations used to optimize the regimen, average fluid consumption was approximately 4.5–5.5 mL per mouse per day. Because SMY is a multicomponent commercial formulation, component-specific oral exposure (mg/kg) was not calculated. Control mice received water. All procedures were approved by the Institutional Animal Care and Use Committee of Nantong University (Approval No. S20250820-001, approved on 20 August 2025) and were conducted in accordance with the Guide for the Care and Use of Laboratory Animals.

### 2.2. Erythrocyte Isolation and In Vitro Treatments

Blood was collected into heparinized tubes from the retro-orbital venous plexus. Samples were centrifuged at 500× *g* for 5 min, and the plasma and buffy coat were removed. Erythrocytes were washed twice with phosphate-buffered saline (PBS). Equal quantities of washed erythrocytes were exposed to 1% O_2_ for 3, 6, or 12 h in a hypoxic workstation (Invivo2400, Ruskinn Technologies, Bridgend, UK) or incubated with PTIO, H_2_O_2_, DPPH, SMY, or vitamin C (VC), as specified for each experiment. PTIO was tested over 0.35–3500 µmol/L; H_2_O_2_ and DPPH were tested over the concentration ranges shown in Figure S1. For all subsequent SMY protection and reversal experiments, PTIO was used at 3500 µmol/L, based on the concentration-response experiment SMY was evaluated at dilutions from 1:50 to 1:2000, with a 1:100 dilution used for the principal in vitro protection and reversal experiments. CpG oligodeoxynucleotide (CpG ODN; 1 mmol/L) served as a positive control for echinocyte formation. Morphological and biochemical endpoints were assessed after the indicated treatments.

### 2.3. Measurement of Osmolality

The osmolality of all treatment solutions (including PTIO, H_2_O_2_, DPPH, and SMY dilutions) was measured using a VAPRO^®^ 5600 vapor pressure osmometer (ELITechGroup, Logan, UT, USA). The instrument was calibrated with standard solutions before each measurement. Briefly, 20 µL of each sample was loaded onto a sample disc and inserted into the sample chamber. Each condition was measured in triplicate at room temperature, and the mean value was used for analysis. Osmolality was expressed as mOsmol/kg.

### 2.4. Blood Gas and Serum Biochemistry

Arterial blood was collected from the left ventricle into blood-gas syringes, sealed immediately, and analyzed at the Affiliated Hospital of Nantong University. The measured variables included arterial oxygen partial pressure (PaO_2_), oxygen saturation (SaO_2_), oxyhemoglobin, P50, and the oxygenation index. Serum alanine aminotransferase (ALT), aspartate aminotransferase (AST), total bilirubin (TBIL), direct bilirubin (DBIL), total protein (TP), albumin (ALB), creatinine (SCr), blood urea nitrogen (BUN), and uric acid (UA) were measured using routine clinical chemistry methods at the same institution.

### 2.5. Erythrocyte Morphology and Scanning Electron Microscopy

A drop of washed erythrocyte suspension was spread as a thin layer on a glass slide and imaged using an upright microscope (DM4000B, Leica, Wetzlar, Germany). For scanning electron microscopy, erythrocyte pellets were fixed overnight at 4 °C in 2.5% glutaraldehyde (G810413, Macklin, Shanghai, China) at a 1:20 sample-to-fixative ratio, washed with double-distilled water, placed on silicon wafers, and vacuum-dried overnight at 37 °C. Samples were imaged using a field-emission scanning electron microscope (Gemini SEM 300, ZEISS, Oberkochen, Germany). Echinocytes were expressed as a percentage of total erythrocytes counted in the analyzed fields.

### 2.6. Chemical Profiling and Cell-Free Antioxidant Assays of SMY

SMY was chemically profiled by ultra-high-performance liquid chromatography-tandem mass spectrometry (UHPLC-MS/MS) by Qingdao Standard Testing Co., Ltd. (Qingdao, China). Total ion chromatograms were acquired in positive- and negative-ion modes, and major signals were tentatively assigned to characteristic ginsenosides and lignans. The pH of serial SMY dilutions was measured with a pH meter. For the DPPH radical-scavenging assay, 20 µL of SMY was mixed with 180 µL of 0.1 mmol/L DPPH (D807297, Macklin, China), incubated at 37 °C in the dark for 30 min, and read at 517 nm. Hydroxyl-radical-scavenging activity was assessed in a Fenton-type system containing 1 mmol/L FeSO_4_, 5 mmol/L H_2_O_2_, and SMY; after 15 min at room temperature, 0.25 mmol/L methylene blue was added and absorbance was measured at 664 nm. Superoxide-related scavenging activity was assessed using a riboflavin/methionine/nitroblue tetrazolium (NBT) photoreduction system containing 0.05 mmol/L NBT, 13 mmol/L L-methionine, and 20 µmol/L riboflavin in PBS. Samples were illuminated for 30 min at room temperature with an LED source positioned 20 cm above the plate, and absorbance was measured at 560 nm; non-illuminated samples served as negative controls. A WST-1-based superoxide dismutase assay kit (S311, Dojindo, Kumamoto, Japan) was used as an additional cell-free assay. Absorbance and fluorescence were recorded using a Synergy 2 microplate reader (BioTek, Winooski, VT, USA).

### 2.7. Flow-Cytometric Measurement of Oxygen-Sensitive RDPP Fluorescence

Erythrocytes were counted, and approximately 1 × 10^6^ cells were aliquoted into labeled microcentrifuge tubes. Cells were centrifuged at 500× *g* for 5 min, and the supernatant was removed. Each sample was resuspended in 100 µL of RDPP oxygen-sensitive probe solution (J&K Scientific, cat. no. 241557, Beijing, China; final concentration, 4 µg/mL) and incubated at 37 °C for 45 min in the dark. After incubation, cells were washed twice with PBS to remove unbound probe and resuspended in PBS for flow-cytometric analysis. Fluorescence was detected in the FL3 channel (488 nm excitation/620 nm emission) using a Gallios flow cytometer (Beckman Coulter, Brea, CA, USA). A total of 10,000 events were acquired per sample, and mean fluorescence intensity (MFI) was analyzed using FlowJo software (v10.8; BD Biosciences, San Jose, CA, USA). Because RDPP fluorescence is quenched by molecular oxygen, higher MFI corresponds to a lower oxygen-associated signal. Accordingly, these measurements were interpreted as relative oxygen-sensitive fluorescence rather than as a direct measurement of intracellular oxygen concentration.

### 2.8. Redox and Hemoglobin Measurements

Blood collected for serum was allowed to clot for 30 min at room temperature and centrifuged at 1000× *g* for 10 min at 4 °C. Serum was collected for immediate analysis. Dihydroethidium (DHE)-derived fluorescence was measured by mixing serum 1:1 with 10 µmol/L DHE (S0063, Beyotime, Shanghai, China), incubating for 30 min in the dark, and reading fluorescence with a microplate reader (Synergy 2, BioTek, Winooski, VT, USA). Malondialdehyde (MDA; BC0025), cysteine (Cys; BC0185), glutathione (GSH; BC1175), oxidized glutathione (GSSG; BC1185), and nitric oxide (NO; BC5480) were measured using commercial kits from Solarbio (Beijing, China). Oxyhemoglobin (OxyHb) and methemoglobin (MetHb) were measured using the Solarbio BC5600 kit according to the manufacturer’s instructions. Superoxide dismutase activity was measured using kit A001-1-1 (Nanjing Jiancheng, Nanjing, China), according to the manufacturer’s instructions. Hydroxyl-radical-scavenging capacity was measured using the methylene-blue/Fenton method described above. For the in vitro hypoxia experiments, the same GSH and GSSG assays were applied to erythrocyte samples collected at 3, 6, and 12 h according to the manufacturers’ instructions. Total glutathione (T-GSH) was calculated as GSH + 2 × GSSG, and the GSSG/GSH ratio was calculated as an index of glutathione redox balance.

### 2.9. Erythrocyte Membrane Preparation

Erythrocyte membranes were prepared by hypotonic lysis. A 7.5 mmol/L phosphate buffer (pH 7.4) was prepared from Na_2_HPO_4_·12H_2_O and NaH_2_PO_4_·2H_2_O. Washed erythrocytes were mixed with lysis buffer at a 1:50 volume ratio in the presence of a protease inhibitor (42932600, Roche, Basel, Switzerland), incubated at 4 °C for 1 h, and centrifuged at 15,000× *g* for 15 min at 4 °C. Membrane pellets were repeatedly washed with hypotonic buffer until the supernatant was clear and were then collected for protein analysis.

### 2.10. Western Blotting

Protein samples were prepared in RIPA buffer (ab156034, Abcam, Cambridge, UK) containing protease inhibitors, and concentrations were determined using a bicinchoninic acid assay (U10007A, UUBIO, Suzhou, China). Proteins (20 µg per lane) were separated by SDS-PAGE and transferred to polyvinylidene fluoride membranes. Membranes were incubated with antibodies against ACSL4 (1:500; sc-271800, SCBT, Dallas, TX, USA), GPX4 (1:1000; ab125066, Abcam, Cambridge, USA), xCT (1:1000; 26864-1-AP, Proteintech, Rosemont, IL, USA), beta-actin (1:5000; 66009-1, Proteintech, Rosemont, IL, USA), GAPDH (1:1000; 5174T, Cell Signaling Technology, Danvers, MA, USA), Band 3 (1:1000; 28131-1-AP, Proteintech, Rosemont, IL, USA), and Na^+^/K^+^-ATPase (1:1000; AF1864, Beyotime, Shanghai, China), followed by species-appropriate secondary antibodies. Chemiluminescent signals were acquired using a Tanon 4100 imaging system (Tanon, Shanghai, China), and band densities were quantified with ImageJ 1.54f (National Institutes of Health, Bethesda, MD, USA).

### 2.11. Co-Immunoprecipitation

Co-immunoprecipitation was performed using non-denaturing lysis buffer (P2179S, Beyotime, China), as previously described [41]. Equal amounts of erythrocyte membrane protein were incubated with an anti-Band 3 antibody or species-matched IgG control and protein A/G beads overnight at 4 °C. Immune complexes were washed, eluted in loading buffer, and analyzed by Western blotting for Syk (A2123, ABclonal, Wuhan, China), SHP2 (A19112, ABclonal, Wuhan, China), PTK2B/PYK2 (F1208, Selleck, Shanghai, China), and Band 3. Co-precipitated proteins were normalized to immunoprecipitated Band 3.

### 2.12. Statistical Analysis

Statistical analyses were performed using GraphPad Prism 9.5. Data are presented as the mean ± standard deviation (SD). Paired two-tailed t-tests were used for matched measurements, and unpaired two-tailed *t*-tests were used for comparisons between two independent groups. One-way or two-way analysis of variance followed by Tukey’s multiple-comparison test was used for experiments with more than two groups or factors. A two-sided *p* value < 0.05 was considered statistically significant.

## 3. Results

### 3.1. Acute HH Exposure Disrupts Redox Balance and Induces Erythrocyte Echinocytosis

**Figure 1:**
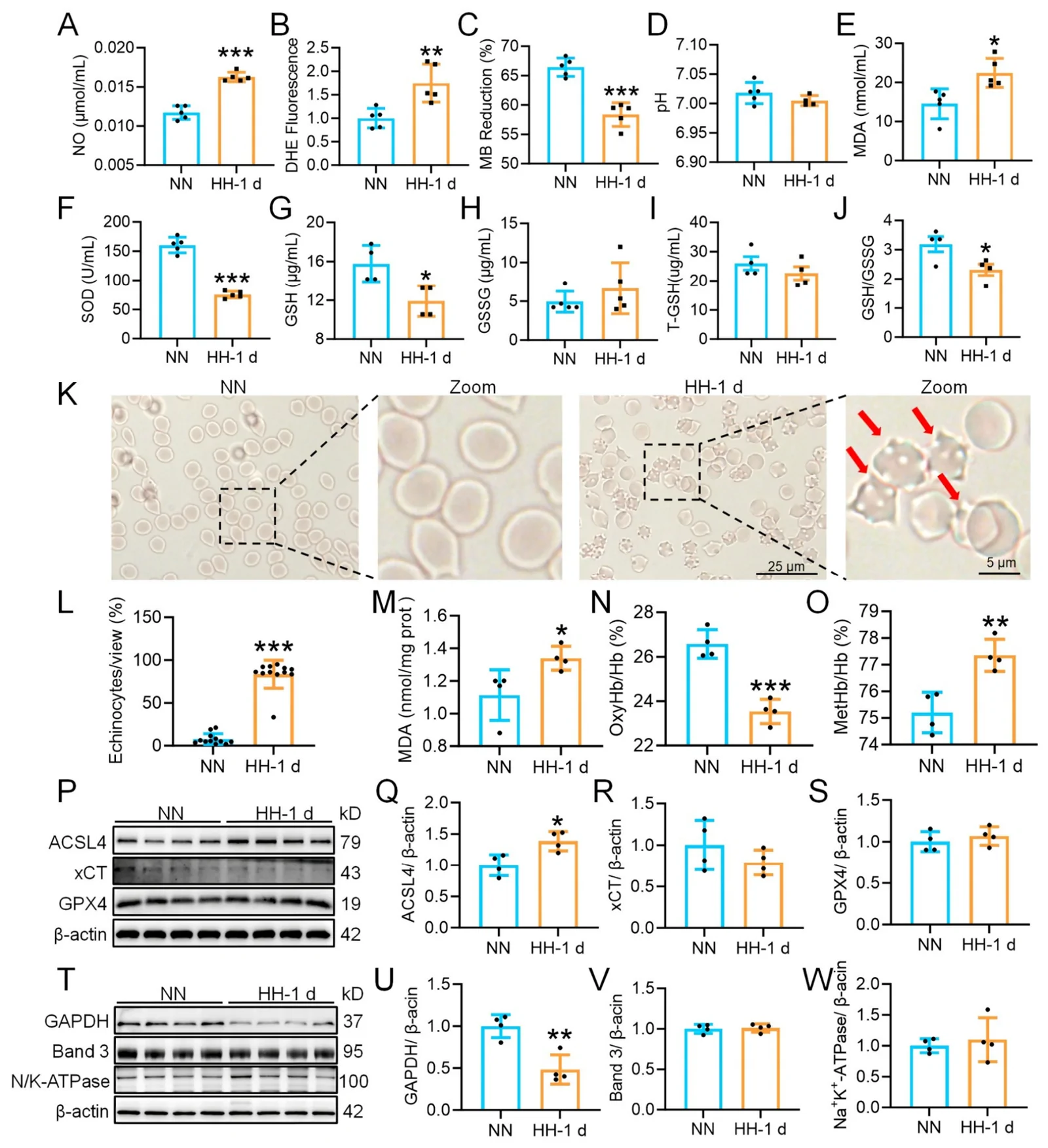
Acute hypobaric hypoxia (HH) disrupts systemic redox homeostasis and induces erythrocyte oxidative injury and echinocytosis. (**A**) Serum nitric oxide (NO). (**B**) Serum dihydroethidium (DHE)-derived fluorescence. (**C**) Hydroxyl-radical-scavenging capacity. (**D**) Blood pH. (**E**) Serum malondialdehyde (MDA). (**F**) Superoxide dismutase (SOD) activity. (**G**) Glutathione (GSH). (**H**) Oxidized glutathione (GSSG). (**I**) Total glutathione (T-GSH; GSH + 2 × GSSG). (**J**) GSH/GSSG ratio. (**K**) Representative light-microscopy images of erythrocytes; red arrows indicate echinocytes in the enlarged HH image. (**L**) Echinocyte quantification. (**M**) Erythrocyte MDA. (**N**,**O**) Oxyhemoglobin (OxyHb) and methemoglobin (MetHb). (**P**,**T**) Representative Western blots. (**Q**–**S**,**U**–**W**) Densitometric analyses of the indicated proteins. Data are presented as mean ± SD. * *p* < 0.05, ** *p* < 0.01, and *** *p* < 0.001 versus the NN group. *n* = 4–6.

Mice were exposed to simulated HH equivalent to 6000 m for 24 h. Compared with NN controls, HH increased serum NO, DHE-derived fluorescence, and MDA, while decreasing hydroxyl-radical-scavenging capacity, SOD activity, and GSH (Figure 1A–C,E–G). GSSG and total glutathione (T-GSH, calculated as GSH + 2 × GSSG) did not differ significantly between groups, whereas the GSH/GSSG ratio was significantly reduced after HH exposure (Figure 1H–J). Blood pH was also unchanged (Figure 1D). Together, these findings indicate that acute HH shifts circulating redox homeostasis toward a more oxidizing state, with the lower GSH/GSSG ratio indicating reduced glutathione redox buffering despite preservation of the measured total glutathione pool.

HH also induced marked erythrocyte echinocytosis and increased erythrocyte MDA (Figure 1K–M). OxyHb decreased, whereas MetHb increased, indicating impaired hemoglobin redox status (Figure 1N,O). ACSL4 abundance increased, while xCT and GPX4 were unchanged (Figure 1P–S). Membrane-associated GAPDH decreased, but total Band 3 and Na^+^/K^+^-ATPase abundance did not change significantly (Figure 1T–W). Thus, the morphological and functional effects of HH were accompanied by lipid peroxidation and selective changes in erythrocyte membrane-associated proteins.

**Figure 2:**
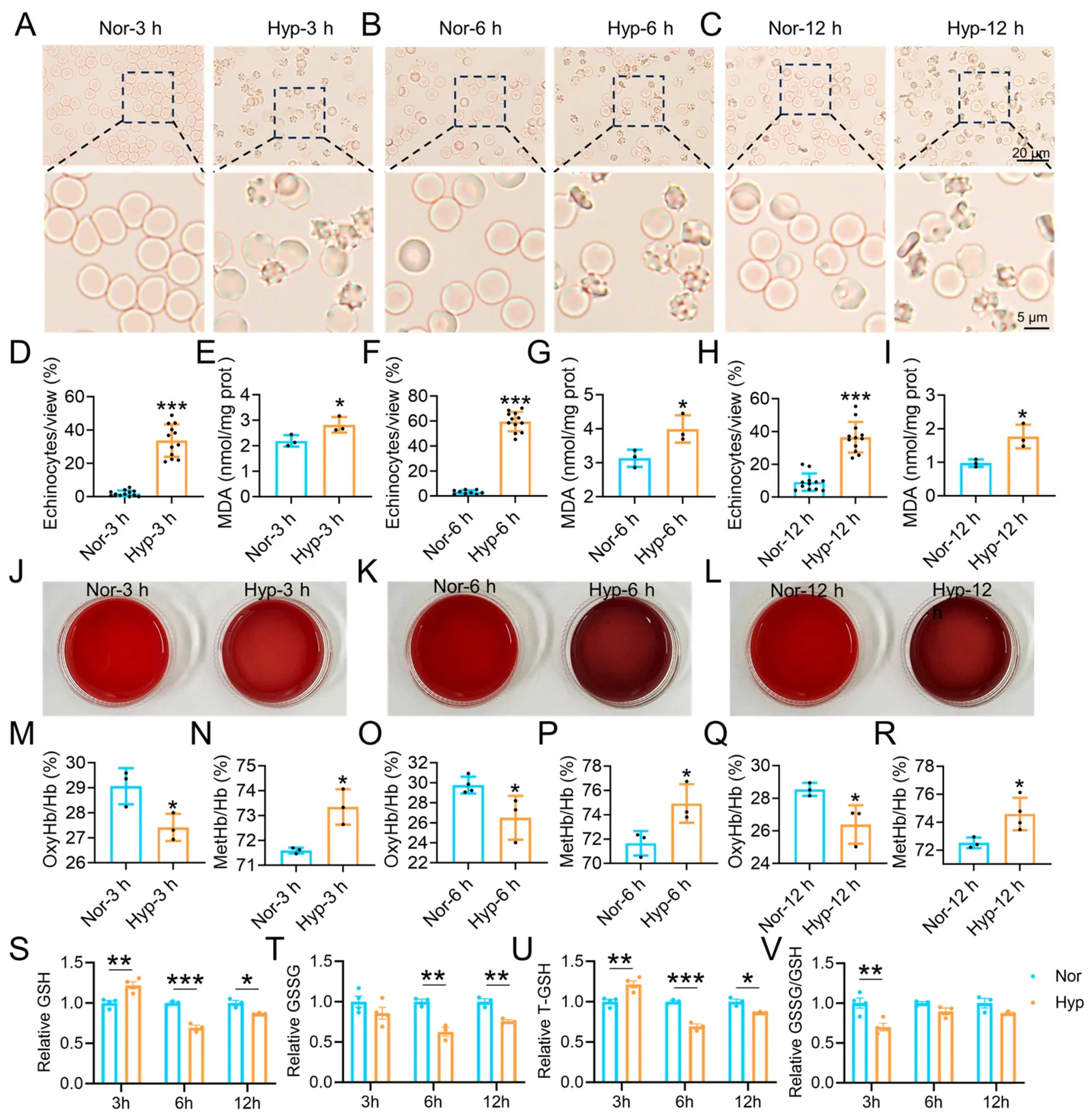
Hypoxic exposure induces erythrocyte echinocytosis, lipid peroxidation, hemoglobin oxidation, and dynamic changes in glutathione homeostasis in vitro. Mouse erythrocytes were exposed to 1% O_2_ for 3, 6, or 12 h. (**A**–**C**) Representative erythrocyte morphology under normoxic (Nor) and hypoxic (Hyp) conditions at 3, 6, and 12 h, respectively. (**D**,**F**,**H**) Echinocyte percentages corresponding to panels A–C. (**E**,**G**,**I**) Erythrocyte MDA levels. (**J**–**L**) Erythrocyte suspension color under Nor or Hyp conditions at 3 h (**J**), 6 h (**K**), and 12 h (**L**). (**M**,**N**) OxyHb and MetHb at 3 h; (**O**,**P**) OxyHb and MetHb at 6 h; (**Q**,**R**) OxyHb and MetHb at 12 h. (**S**–**U**) Relative GSH, GSSG, and T-GSH levels, respectively, normalized to the corresponding normoxic control; (**V**) GSSG/GSH ratio after 3, 6, and 12 h of in vitro exposure to 1% O_2_. Absolute glutathione concentrations and calculated E_h′ values are provided in Supplementary Table S1. T-GSH was calculated as GSH + 2 × GSSG. Data are presented as mean ± SD. * *p* < 0.05, ** *p* < 0.01, and *** *p* < 0.001 versus the corresponding Nor group. *n* = 3–6.

To determine whether hypoxia directly affected erythrocytes, washed cells were exposed to 1% O_2_ in vitro for 3, 6, or 12 h. Hypoxia induced echinocytosis and increased MDA at all examined time points (Figure 2A–I). Erythrocyte suspensions became visibly darker, particularly after 6 and 12 h, and showed lower OxyHb and higher MetHb (Figure 2J–R). These findings demonstrate that hypoxia can directly induce erythrocyte lipid peroxidation, morphological deformation, and hemoglobin oxidation.

We next examined temporal changes in glutathione homeostasis during hypoxic exposure (Supplementary Table S1 and Figure 2S–V). After 3 h at 1% O_2_, erythrocytes showed a transient reductive adaptation, characterized by increases in GSH and T-GSH, a lower GSSG/GSH ratio, and a more negative formal glutathione redox potential (E_h′; −196.59 ± 0.70 mV vs. −189.47 ± 0.95 mV in normoxia). This response was not sustained. At 6 h, GSH and T-GSH declined, the GSSG/GSH ratio returned toward the normoxic level, and E_h′ became less negative than in normoxia (−175.67 ± 0.23 vs. −178.95 ± 0.12 mV), consistent with loss of the early reducing response. By 12 h, GSH and T-GSH were further depleted, whereas the GSSG/GSH ratio and E_h′ were similar between normoxic and hypoxic erythrocytes (−201.94 ± 0.11 vs. −202.06 ± 1.15 mV). Thus, ratio-based redox indices did not fully reflect the progressive depletion of the glutathione pool at later time points and should be interpreted together with absolute glutathione concentrations and independent markers of oxidative injury.

### 3.2. PTIO Recapitulates HH-Associated Echinocytosis In Vitro

To identify an operational in vitro redox challenge that reproduced the characteristic erythrocyte morphology observed after HH, we compared PTIO, H_2_O_2_, and DPPH, with CpG ODN as a positive control for echinocyte formation. Echinocytosis was the primary selection criterion; changes in hemoglobin redox indices were considered supportive rather than used to establish a biochemical match to HH. Across the tested concentration ranges, none of the treatments produced changes in pH or osmolality large enough to independently explain erythrocyte crenation. PTIO produced the most pronounced HH-like echinocytosis and was therefore selected for subsequent mechanistic and SMY intervention experiments; its concomitant decrease in OxyHb and increase in MetHb provided additional phenotypic similarity to HH.

**Figure 3:**
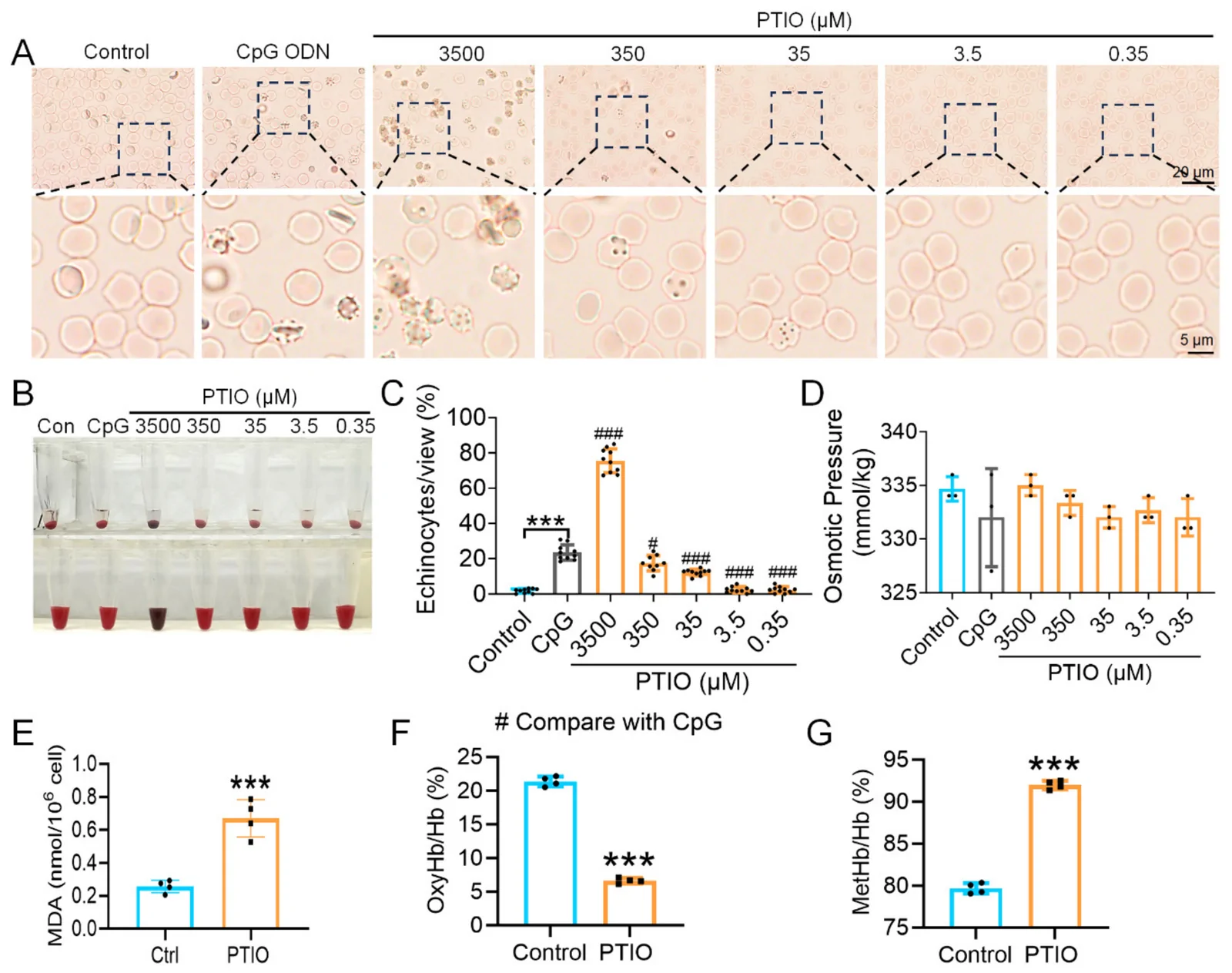
PTIO induces echinocytosis and impairs hemoglobin redox status. (**A**) Representative erythrocyte morphology after exposure to the indicated PTIO concentrations; CpG oligodeoxynucleotide (CpG ODN) was used as a positive control for echinocytosis. (**B**) Erythrocyte pellets and suspensions. (**C**) Echinocyte percentages. (**D**) Osmolality of PTIO-containing solutions. (**E**–**G**) Effects of 3500 µmol/L PTIO on erythrocyte MDA, OxyHb, and MetHb. Data are presented as mean ± SD. and *** *p* < 0.001 versus control; # *p* < 0.05 and ### *p* < 0.001 for the indicated comparisons. *n* = 4–6. PTIO, 2-phenyl-4,4,5,5-tetramethylimidazoline-1-oxyl 3-oxide.

PTIO induced concentration-dependent echinocytosis and increased MDA, accompanied by lower OxyHb and higher MetHb (Figure 3A–G). H_2_O_2_ also induced echinocytosis but produced less consistent changes in hemoglobin indices, whereas DPPH did not significantly alter erythrocyte morphology or hemoglobin status under the tested conditions (Figure S1). These comparisons support the use of PTIO as a morphology-based operational redox challenge for the subsequent SMY experiments.

### 3.3. SMY Exhibits Antioxidant Activity and Prevents PTIO-Induced Erythrocyte Injury

**Figure 4:**
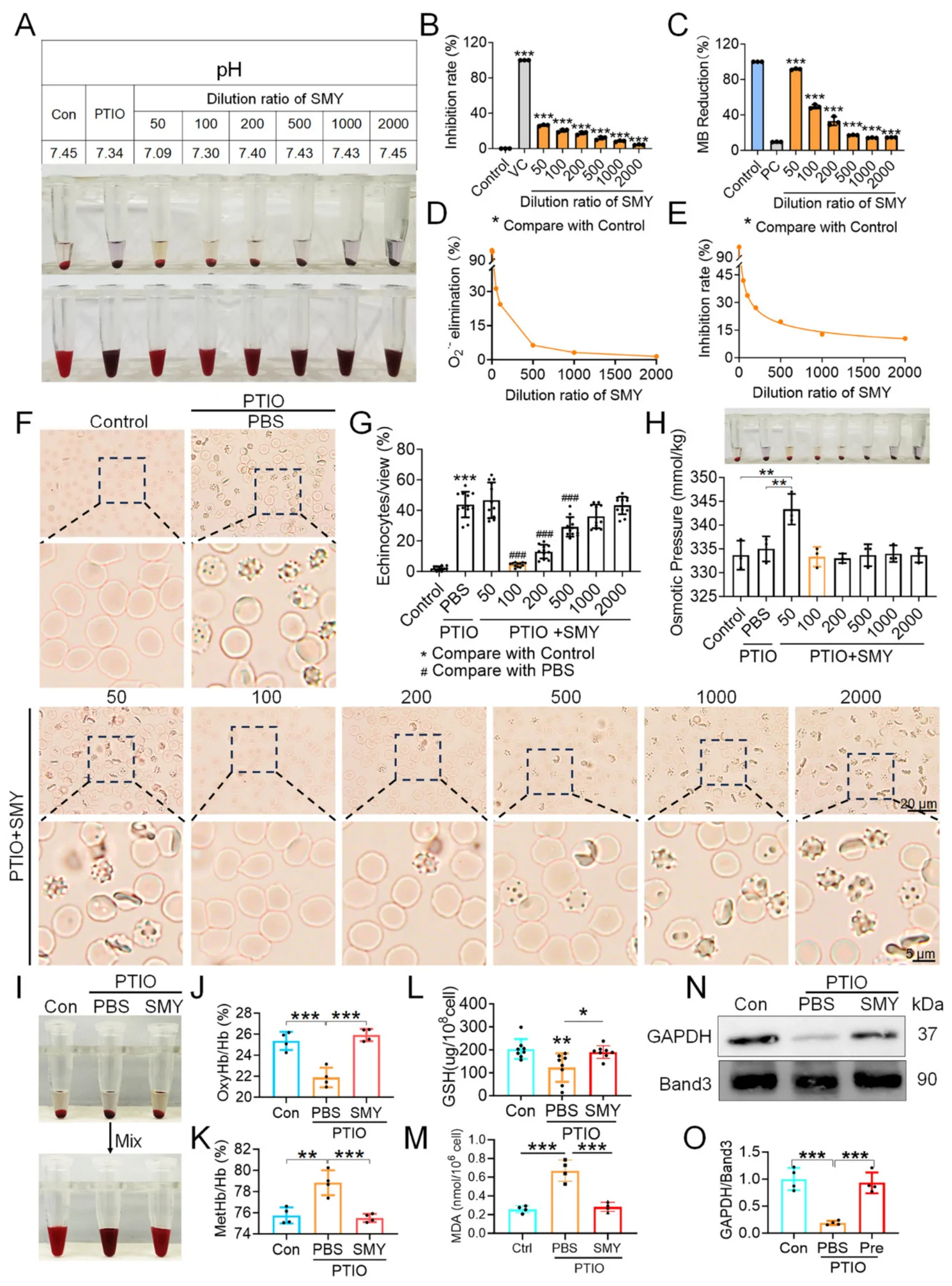
Shengmai Yin (SMY) exhibits cell-free antioxidant activity and prevents 3500 µmol/L PTIO-induced erythrocyte injury. (**A**) pH of serial SMY dilutions. (**B**) DPPH radical-scavenging activity. (**C**) Hydroxyl-radical-scavenging activity measured by methylene-blue reduction. (**D**) Superoxide-related scavenging activity in the nitroblue tetrazolium (NBT) photoreduction assay. (**E**) WST-1-based superoxide-related assay. (**F**) Representative erythrocyte morphology after PTIO exposure with the indicated SMY dilutions. (**G**) Echinocyte percentages. (**H**) Osmolality. (**I**) Erythrocyte pellets and suspensions. (**J**,**K**) OxyHb and MetHb. (**L**,**M**) GSH and MDA. (**N**) Representative Western blot of membrane-associated GAPDH and Band 3. (**O**) GAPDH/Band 3 densitometry. SMY is expressed as the *v*/*v* dilution of the commercial stock formulation; larger dilution factors therefore correspond to lower SMY concentrations. Data are presented as mean ± SD. * *p* < 0.05, ** *p* < 0.01, and *** *p* < 0.001 versus control; ### *p* < 0.001 for the indicated comparison. n = 4–6. Orange color bar in subfigure G and H indicates the 100 µM group, which was selected for the subsequent mechanistic experiments based on these results.

UHPLC-MS/MS profiling of SMY detected major chromatographic signals tentatively assigned to schisandrin-related lignans and several ginsenosides, including Rg1, Rg2, Rg3, and Rf (Figure S2). Serially diluted SMY was weakly acidic at high concentrations and approached neutral pH at dilutions of 1:50 or greater (Figure 4A). SMY reduced DPPH radical, hydroxyl-radical, and superoxide-related readouts in cell-free assays in a concentration-dependent manner (Figure 4B–E).

SMY prevented H_2_O_2_- and PTIO-induced echinocytosis, with the strongest protection observed at a 1:100 dilution under the tested conditions (Figure 4F–H and Figure S3). In PTIO-treated erythrocytes, SMY limited darkening of the cell suspension, preserved OxyHb, reduced MetHb, attenuated GSH depletion and MDA accumulation, and maintained membrane-associated GAPDH (Figure 4I–O). These results indicate that SMY protects erythrocytes from PTIO-induced oxidative and functional injury.

### 3.4. SMY Prevents Hypoxia- and HH-Induced Erythrocyte Dysfunction

**Figure 5:**
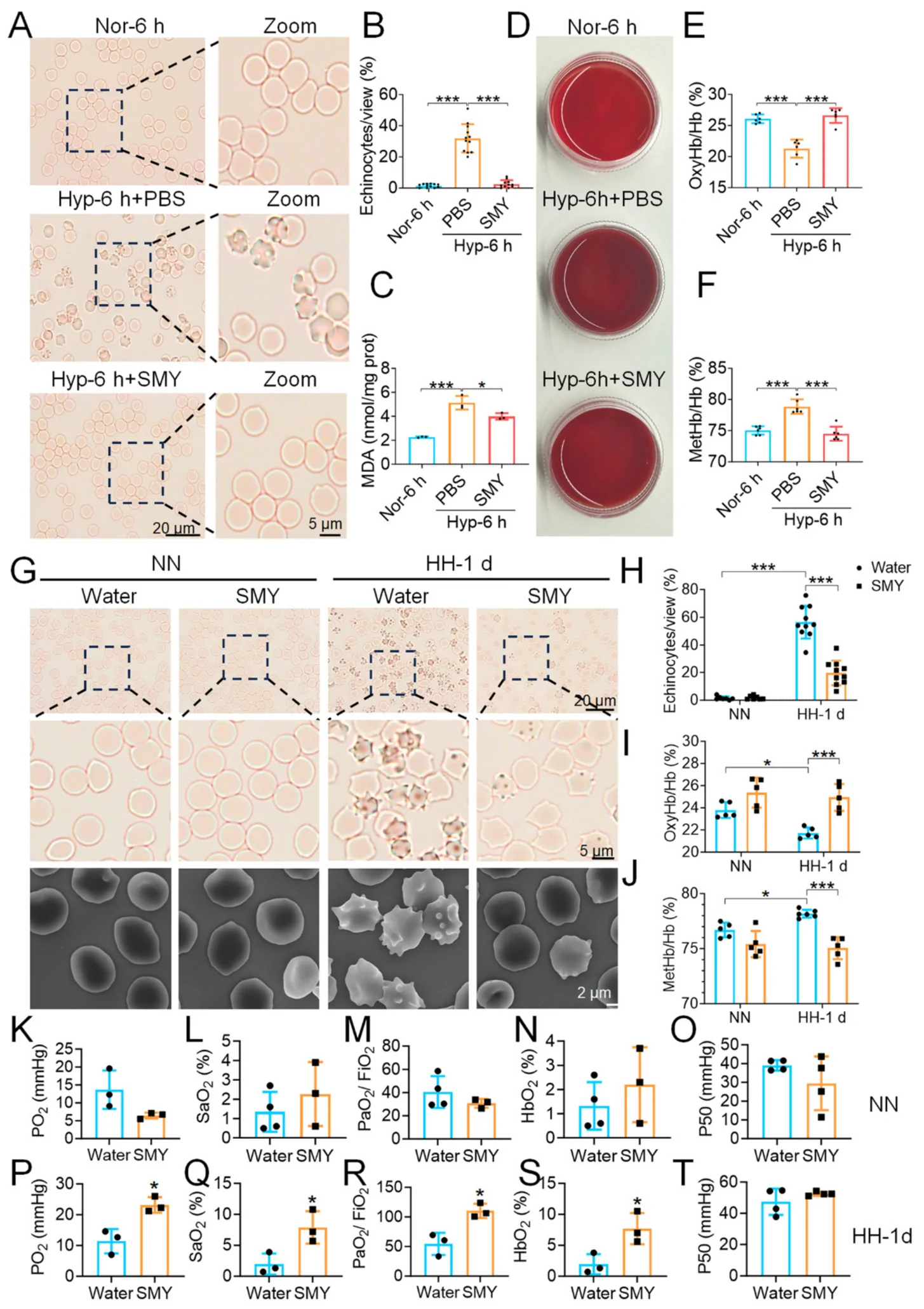
SMY prevents hypoxia- and HH-induced erythrocyte injury and improves arterial oxygenation. Washed erythrocytes were exposed to 1% O_2_ for 6 h with vehicle or SMY. (**A**) Representative morphology. (**B**) Echinocyte percentages. (**C**) Erythrocyte MDA. (**D**) Suspension color. (**E**,**F**) OxyHb and MetHb. For the in vivo experiment, mice received water or a 1:30 SMY preparation for 3 days before 24 h of HH. (**G**) Representative light- and scanning-electron-microscopy images. (**H**) Echinocyte percentages. (**I**,**J**) Erythrocyte OxyHb and MetHb. (**K**,**P**) PaO_2_. (**L**,**Q**) SaO_2_. (**M**,**R**) Oxygenation index. (**N**,**S**) Arterial oxyhemoglobin. (**O**,**T**) P50. Data are presented as mean ± SD. * *p* < 0.05 and *** *p* < 0.001 versus the corresponding Nor, NN, or indicated group. *n* = 4–6.

In washed erythrocytes exposed to 1% O_2_ for 6 h, SMY pretreatment reduced echinocytosis and MDA accumulation (Figure 5A–C). SMY also preserved the bright-red appearance of the erythrocyte suspension, increased OxyHb, and reduced MetHb relative to hypoxic vehicle-treated cells (Figure 5D–F).

In vivo, mice received the 1:30 SMY drinking-water preparation for 3 days before 24 h of HH. To evaluate short-term tolerability under the experimental conditions, we assessed serum biochemical indices and organ histology. SMY did not significantly alter serum ALT, AST, TBIL, DBIL, TP, ALB, SCr, BUN, or UA (Figure S4A–I), and hematoxylin and eosin staining revealed no overt pathological changes in the heart, liver, spleen, lung, or kidney (Figure S4J). These findings indicate no detectable short-term hepatic, renal, or histological toxicity by the measured endpoints. We next assessed the effects of SMY on HH-induced erythrocyte injury. SMY reduced the proportion of circulating echinocytes, increased OxyHb, and decreased MetHb in HH-exposed mice (Figure 5G–J). SMY also increased PaO_2_, SaO_2_, the oxygenation index, and arterial oxyhemoglobin under HH (Figure 5P–S). It did not significantly alter these variables under NN conditions and did not change P50 in either condition (Figure 5K–O,T). Thus, prophylactic SMY attenuated erythrocyte injury and was associated with improved arterial oxygenation during acute HH.

### 3.5. SMY Reverses Established Echinocytosis and Improves Hemoglobin Redox Status

**Figure 6:**
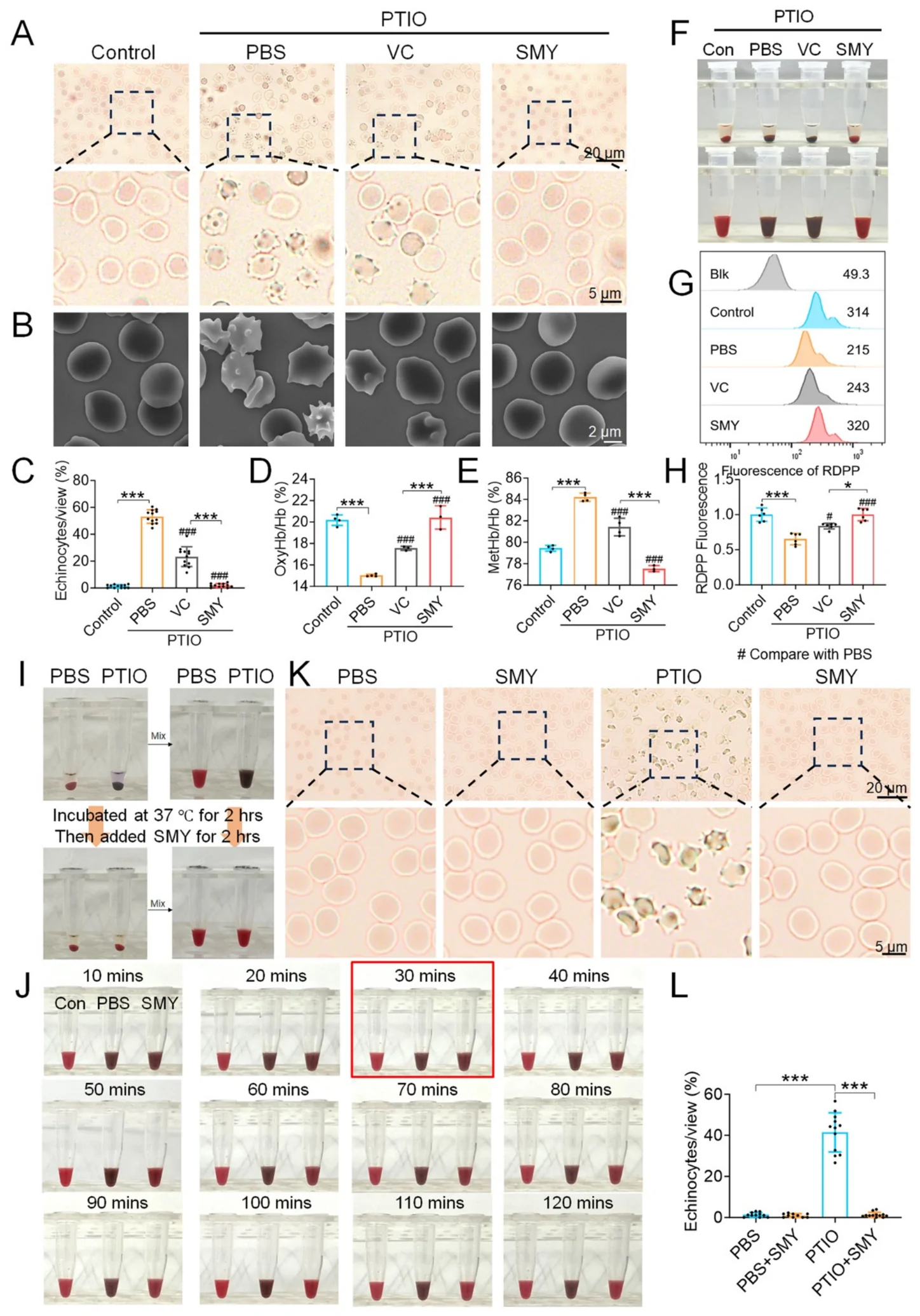
SMY reverses established PTIO-induced echinocytosis and improves hemoglobin redox indices. Erythrocytes were first exposed to PTIO and subsequently treated with vehicle, vitamin C (VC), or SMY. (**A**,**B**) Representative light- and scanning-electron-microscopy images. (**C**) Echinocyte percentages. (**D**,**E**) OxyHb and MetHb. (**F**) Erythrocyte suspension color. (**G**) Representative flow-cytometry profiles of oxygen-associated fluorescence. (**H**) Mean fluorescence intensity. (**I**) Experimental timeline. (**J**) Suspension color during SMY treatment. (**K**) Representative morphology before and after SMY. (**L**) Echinocyte percentages. Data are presented as mean ± SD. * *p* < 0.05, and *** *p* < 0.001 for the indicated comparisons. # *p* < 0.05 and ### *p* < 0.001 versus PBS. *n* = 4–6. Red box ndicates that the color change became obvious from 30 min onward, suggesting that the treatment began to take effect.

To assess reversal rather than prevention, erythrocytes were first exposed to PTIO and then treated with SMY or VC. Both antioxidants reduced echinocytosis, increased OxyHb, decreased MetHb, and lowered the oxygen-associated fluorescence signal measured by flow cytometry (Figure 6A–H). Under the tested conditions, SMY produced greater recovery than VC for several morphological and hemoglobin endpoints.

Time-course imaging showed that SMY changed the erythrocyte suspension from dark red toward bright red and significantly reduced the proportion of echinocytes within 30 min (Figure 6I–L). These observations indicate that SMY can rapidly reverse established PTIO-induced morphological changes, while improving hemoglobin redox indices over the treatment interval.

### 3.6. SMY Normalizes Band 3 Interactions with Syk and SHP2

**Figure 7:**
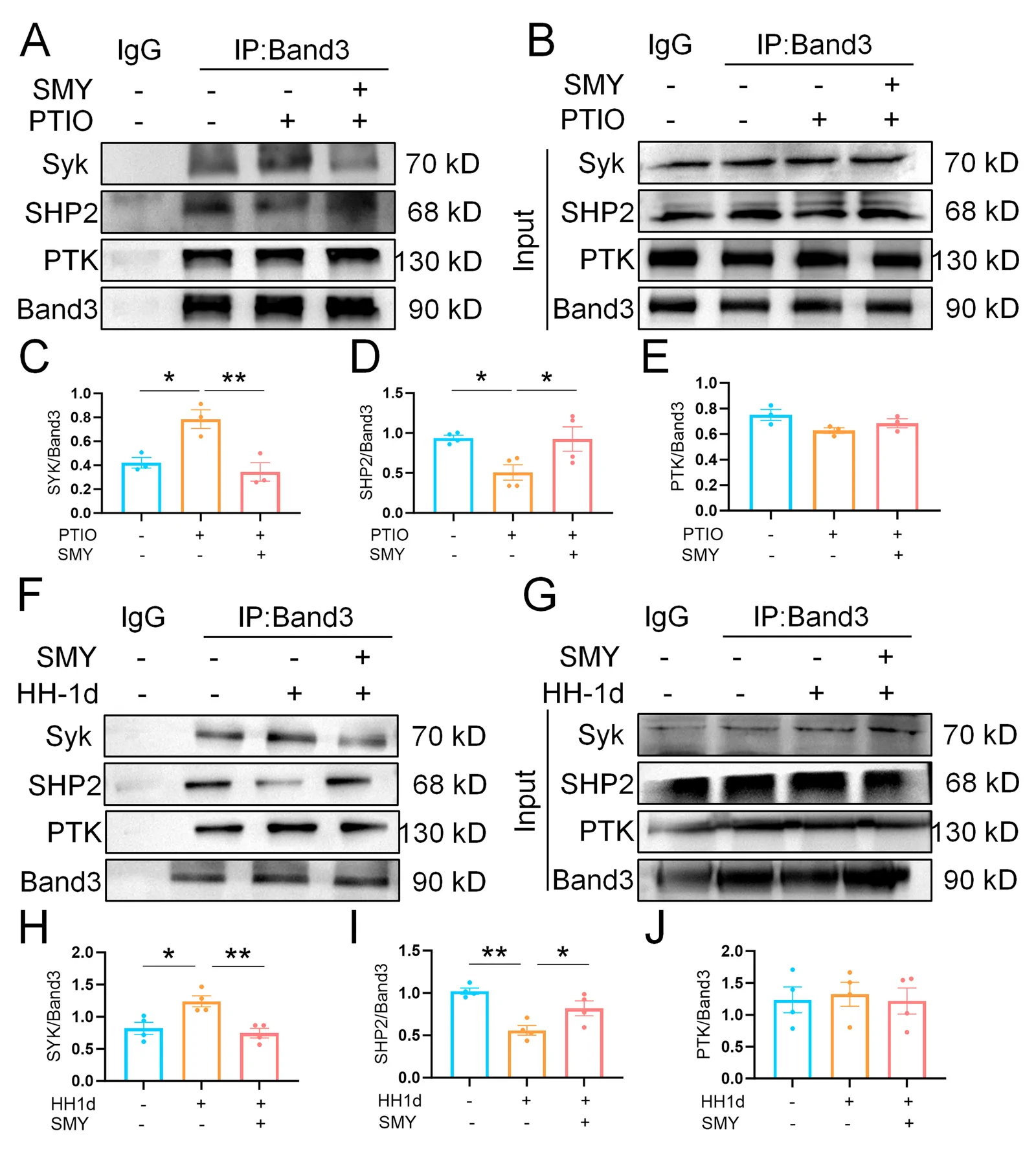
SMY normalizes Band 3 interactions with Syk and SHP2. (**A**,**B**) Co-immunoprecipitation of Band 3-containing erythrocyte membrane complexes after PTIO exposure with or without SMY. (**C**–**E**) Quantification of Band 3-associated Syk, SHP2, and PTK2B, respectively. (**F**,**G**) Co-immunoprecipitation after HH exposure with or without SMY. (**H**–**J**) Quantification of Band 3-associated Syk, SHP2, and PTK2B, respectively. Co-precipitated proteins were normalized to immunoprecipitated Band 3. Data are presented as mean ± SD. * *p* < 0.05 and ** *p* < 0.01 for the indicated comparisons. *n* = 4–6.

Band 3-containing membrane complexes were analyzed by co-immunoprecipitation. PTK2B association with Band 3 was not significantly altered by PTIO, HH, or SMY (Figure 7A,E,F,J). By contrast, PTIO and HH increased the association of Syk with Band 3, and SMY reduced this interaction toward control levels (Figure 7B,C,G,H). PTIO and HH also decreased the association of SHP2 with Band 3, whereas SMY restored SHP2 binding (Figure 7B,D,G,I). These results show that SMY normalizes reciprocal changes in Band 3 association with a tyrosine kinase and phosphatase during oxidative and hypoxic stress.

## 4. Discussion

The present study identifies acute erythrocyte injury as a prominent component of the systemic response to HH and shows that SMY attenuates this injury. A 24 h exposure equivalent to 6000 m shifted circulating redox indices toward oxidation, increased erythrocyte lipid peroxidation, induced echinocytosis, and promoted conversion of OxyHb to MetHb. These changes were reproduced directly by 1% O_2_ in isolated erythrocytes, indicating that hypoxia can act on erythrocytes independently of other organ systems. SMY reduced oxidative readouts, preserved erythrocyte morphology and hemoglobin status, improved arterial oxygenation, and normalized stress-associated changes in Band 3–Syk/SHP2 interactions.

The increase in erythrocyte MDA and ACSL4 abundance is consistent with enhanced membrane lipid peroxidation. ACSL4 promotes incorporation of polyunsaturated fatty acids into phospholipids that are susceptible to oxidation [43], whereas the xCT-GSH-GPX4 system limits lipid peroxide accumulation [44]. In this acute model, xCT and GPX4 abundance did not change, suggesting that the measured phenotype was associated more closely with increased oxidative burden than with loss of these proteins. Because mature erythrocytes lack de novo protein synthesis, the higher ACSL4 signal should not be interpreted as transcriptional upregulation; it may reflect altered membrane association, differences in circulating erythrocyte subpopulations, or changes in protein stability. Direct lipidomic analysis would be required to define the oxidized phospholipid species involved.

The temporal dissociation between glutathione indices and oxidative injury markers provides insight into the progression of hypoxic erythrocyte injury. The return of the GSSG/GSH ratio toward the normoxic level at 6 and 12 h did not indicate recovery; rather, it occurred as the total glutathione pool progressively declined. At 6 h, E_h′ was less negative under hypoxia than under normoxia (−175.67 vs. −178.95 mV), consistent with loss of the early reductive response. By 12 h, however, both the GSSG/GSH ratio and E_h′ were similar between groups despite marked GSH and T-GSH depletion. The calculated E_h′ values are broadly comparable to values reported in mammalian erythrocytes [45], with modest differences potentially attributable to species and experimental conditions. Meanwhile, MDA and MetHb continued to accumulate, indicating that the transient increase in GSH at 3 h was insufficient to prevent ongoing lipid and hemoglobin oxidation. Together, these findings show that neither the GSSG/GSH ratio nor E_h′ should be interpreted in isolation when the absolute glutathione pool is changing substantially; integration with GSH/T-GSH concentrations and independent oxidative-injury markers provides a more complete assessment of erythrocyte redox status.

Band 3 is a central organizer of erythrocyte membrane structure and metabolism. Its interaction with ankyrin is oxygen sensitive [16], and HH-reoxygenation can impair its transport and protein-binding functions [17]. Total Band 3 abundance was unchanged in the present study, but membrane-associated GAPDH decreased. This result is compatible with oxygen-dependent displacement of glycolytic enzymes from Band 3 and increased glycolytic flux during hypoxia [46]. At the same time, lower OxyHb and higher MetHb indicate oxidation of hemoglobin iron from Fe^2+^ to Fe^3+^, which prevents oxygen binding. Although changes in hemoglobin concentration and oxygen affinity contribute to longer-term acclimatization [47,48], the acute increase in MetHb observed here represents a potentially maladaptive impairment of oxygen transport.

PTIO was the only tested redox challenge that reproduced both marked echinocytosis and hemoglobin oxidation. However, PTIO is a nitric oxide scavenger and redox-active nitroxide rather than a selective superoxide donor [26]. In addition, bulk DHE fluorescence is sensitive to several oxidation products and does not by itself provide definitive molecular identification of superoxide. The data therefore support involvement of a superoxide-associated disturbance in nitric oxide/redox balance, but they do not establish superoxide as the sole causal species. Confirmation with orthogonal methods, such as chromatographic separation of DHE oxidation products, electron paramagnetic resonance, selective enzymatic scavengers, or genetic redox interventions, would strengthen this mechanistic conclusion. Importantly, the PTIO system should not be considered equivalent to HH; rather, it serves as a controlled operational redox challenge that reproduces selected erythrocyte phenotypes and complements the integrated in vivo HH model. Oxidant-challenge/antioxidant-rescue paradigms are well established in RBC research. In H_2_O_2_-treated human RBCs, quercetin attenuated oxidative injury, MetHb formation, and Band 3 dysfunction [27], while a recent review summarizes broader antioxidant strategies targeting RBC redox homeostasis and membrane integrity [28].

SMY reduced multiple cell-free radical readouts and protected erythrocytes against peroxide-, PTIO-, and hypoxia-induced injury. This broad activity is plausible for a complex formulation containing ginsenosides, lignans, and other constituents [37]. Ginseng saponins have previously been reported to improve oxidant-induced erythrocyte deformability and regulate erythrocyte tyrosine phosphorylation and glycolysis [36]. The present results extend those observations to HH and show that SMY can both prevent injury and reverse established echinocytosis. The lack of a P50 change suggests that improved arterial oxygenation was not mediated by a major shift in hemoglobin-oxygen affinity but was associated with preservation of OxyHb and erythrocyte morphology. Because pulmonary ventilation, gas diffusion, and hemodynamics were not directly measured, the mechanism underlying the higher PaO_2_ after SMY cannot be resolved from the present study and should not be attributed solely to erythrocyte effects. The concept of preserving erythrocyte function through redox modulation also aligns with work in transfusion medicine, where oxidative damage, membrane remodeling, and metabolic depletion contribute to the RBC storage lesion [49]. Nemkov et al. further showed that carnitine availability is linked to lipid-damage repair during RBC aging and that L-carnitine supplementation of stored murine RBCs improved post-transfusion recovery [50]. Although the current study concerns acute HH rather than storage-related injury, these shared redox and membrane processes suggest that SMY or its active constituents may warrant future evaluation in RBC preservation models. Nevertheless, the active constituents and their pharmacokinetic exposure remain to be defined, and quantitative batch standardization of the formulation will be important for reproducibility.

The reciprocal changes in Band 3 binding to Syk and SHP2 provide a potential membrane mechanism for the morphological effects of SMY. Tyrosine phosphorylation of Band 3 weakens membrane-cytoskeletal anchoring and promotes membrane reorganization [51,52]. Increased Syk association and decreased SHP2 association during PTIO or HH are therefore consistent with a shift toward Band 3 phosphorylation, whereas SMY restored the interaction pattern toward control conditions. Because Band 3 phosphotyrosine was not measured directly and causality was not tested with Syk or SHP2 manipulation, these co-immunoprecipitation findings should be interpreted as evidence of altered protein association rather than proof that the Syk/SHP2 balance mediates all protective effects of SMY.

Several limitations should be considered. The study used only male mice and a short HH exposure, and the SMY dose consumed per kilogram of body weight was not directly quantified. PTIO and DHE provide indirect evidence regarding the responsible ROS species. The flow-cytometric oxygen-associated signal requires full methodological validation, and the in vitro concentrations may not reflect in vivo exposure. In addition, the short-term biochemical and histological assessment does not establish comprehensive safety. The physiological basis of the SMY-associated increase in PaO_2_ was not directly assessed because ventilatory, pulmonary-diffusion, and hemodynamic measurements were not performed. Future work should include both sexes, dose-exposure analyses, standardized chemical quantification of SMY, direct measurement of Band 3 phosphorylation, selective manipulation of Syk and SHP2, and validation in longer-term or clinically relevant HA models.

## 5. Conclusions

Acute HH induces erythrocyte echinocytosis, lipid peroxidation, hemoglobin oxidation, and altered Band 3 interactions with Syk and SHP2. SMY attenuates these changes, improves arterial oxygenation, and rapidly reverses established echinocytosis in vitro. The findings support a model in which SMY preserves erythrocyte function through broad redox modulation and normalization of Band 3-associated protein interactions. Direct identification of the causal ROS species, active SMY constituents, and Band 3 phosphorylation events will be necessary before clinical translation.

## Data Availability

The data supporting the findings of this study are available from the corresponding author upon reasonable request.

## Supplementary Materials

The following supporting information can be downloaded at https://www.mdpi.com/article/10.3390/antiox15091205/s1, Figure S1, Effects of H_2_O_2_ and DPPH on erythrocyte morphology and hemoglobin redox status; Figure S2, UHPLC-MS/MS total ion chromatograms of SMY in negative-ion (A) and positive-ion (B) modes; Figure S3, SMY prevents H_2_O_2_-induced echinocytosis; Figure S4, Short-term SMY administration produces no detectable hepatic, renal, or histological toxicity. Table S1, Erythrocyte glutathione redox status following in vitro treatment of 1% O_2_.

## Abbreviations

ACSL4	acyl-CoA synthetase long-chain family member 4
ALB	albumin
ALT	alanine aminotransferase
AST	aspartate aminotransferase
BUN	blood urea nitrogen
Co-IP	co-immunoprecipitation
DHE	dihydroethidium
DPPH	2,2-diphenyl-1-picrylhydrazyl
GAPDH	glyceraldehyde-3-phosphate dehydrogenase
GPX4	glutathione peroxidase 4
GSH	glutathione
GSSG	oxidized glutathione
HA	high altitude
HH	hypobaric hypoxia
MDA	malondialdehyde
MetHb	methemoglobin
NBT	nitroblue tetrazolium
NN	normobaric normoxia
NO	nitric oxide
OxyHb	oxyhemoglobin
PBS	phosphate-buffered saline
PTIO	2-phenyl-4,4,5,5-tetramethylimidazoline-1-oxyl 3-oxide
RBC	red blood cell
ROS	reactive oxygen species
SaO_2_	arterial oxygen saturation
SCr	serum creatinine
SHP2	Src homology 2 domain-containing phosphatase 2
SMY	Shengmai Yin
SOD	superoxide dismutase
Syk	spleen tyrosine kinase
TBIL	total bilirubin
TP	total protein
UA	uric acid
VC	vitamin C
