# Supplementary material for: Shengmai Yin Attenuates Hypobaric Hypoxia-Induced Erythrocyte Echinocytosis by Restoring Redox Homeostasis and Normalizing Band 3–Syk/SHP2 Interactions
Source: Antioxidants (Basel). 2026 Sep 20;15(9):1205. doi: 10.3390/antiox15091205 (PMC13603466; doi:10.3390/antiox15091205)
Supplement: Supplementary file 1 [file antioxidants-15-01205-s001.zip › antioxidants-4492576-supplementary.pdf]

# Supplementary Figures

Shengmai Yin Attenuates Hypobaric Hypoxia-Induced Erythrocyte Echinocytosis by Restoring Redox Homeostasis and Normalizing Band 3–Syk/SHP2 Interactions

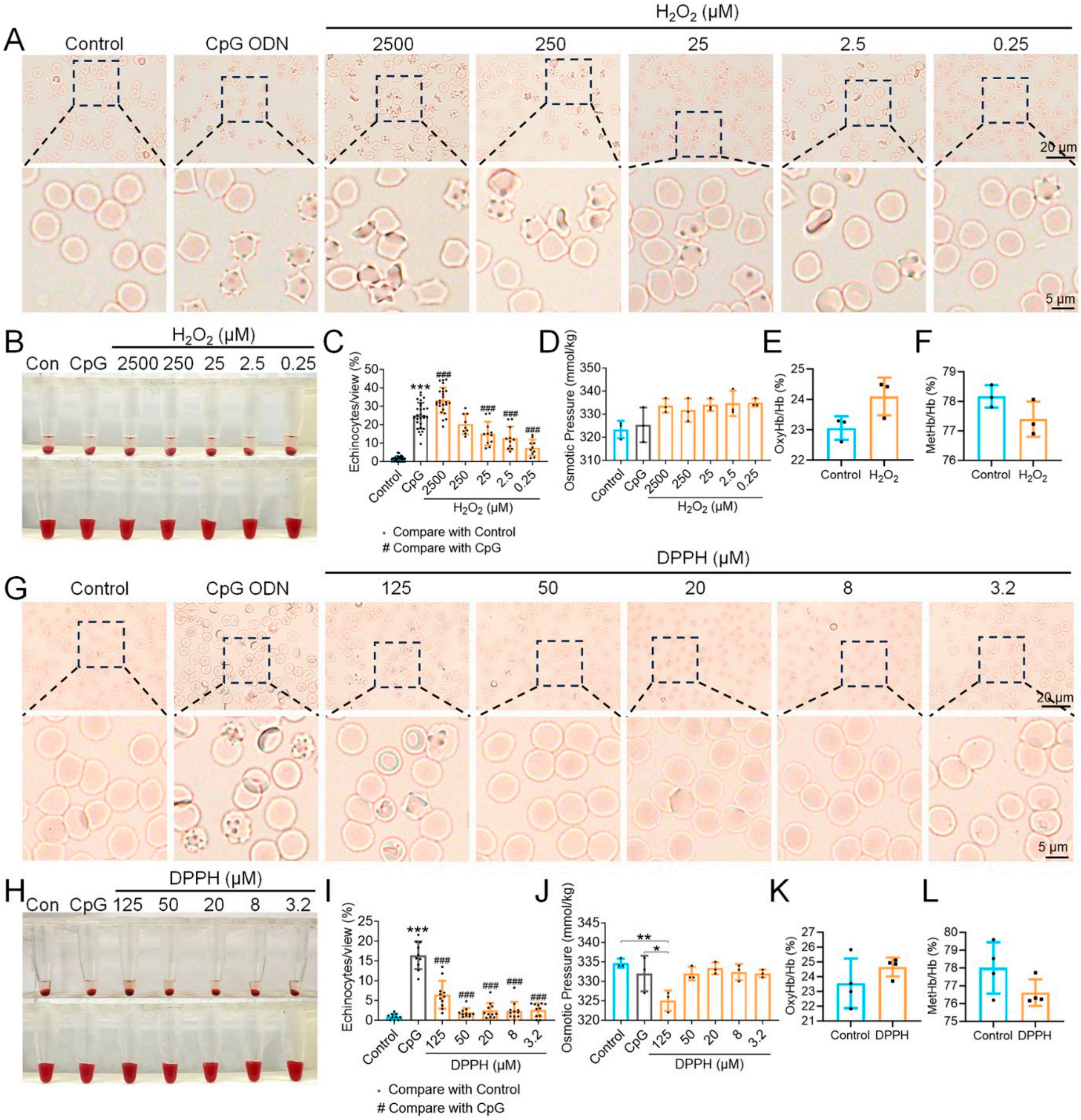


**Figure S1. Effects of $H_2O_2$ and DPPH on erythrocyte morphology and hemoglobin redox status. (A,G) Representative erythrocyte morphology after exposure to the indicated concentrations of $H_2O_2$ or DPPH. (B,H) Erythrocyte pellets and suspensions. (C,I) Echinocyte percentages. (D,J) Osmolality. (E,F) OxyHb and MetHb after 2500 μmol/L $H_2O_2$. (K,L) OxyHb and MetHb after 50 mmol/L DPPH. CpG oligodeoxynucleotide (CpG ODN) was used as a positive control for echinocytosis. Data are presented as mean ± SD. *$p < 0.05$, **$p < 0.01$, and ***$p < 0.001$ versus control; ###$p < 0.001$ for the indicated comparison.**

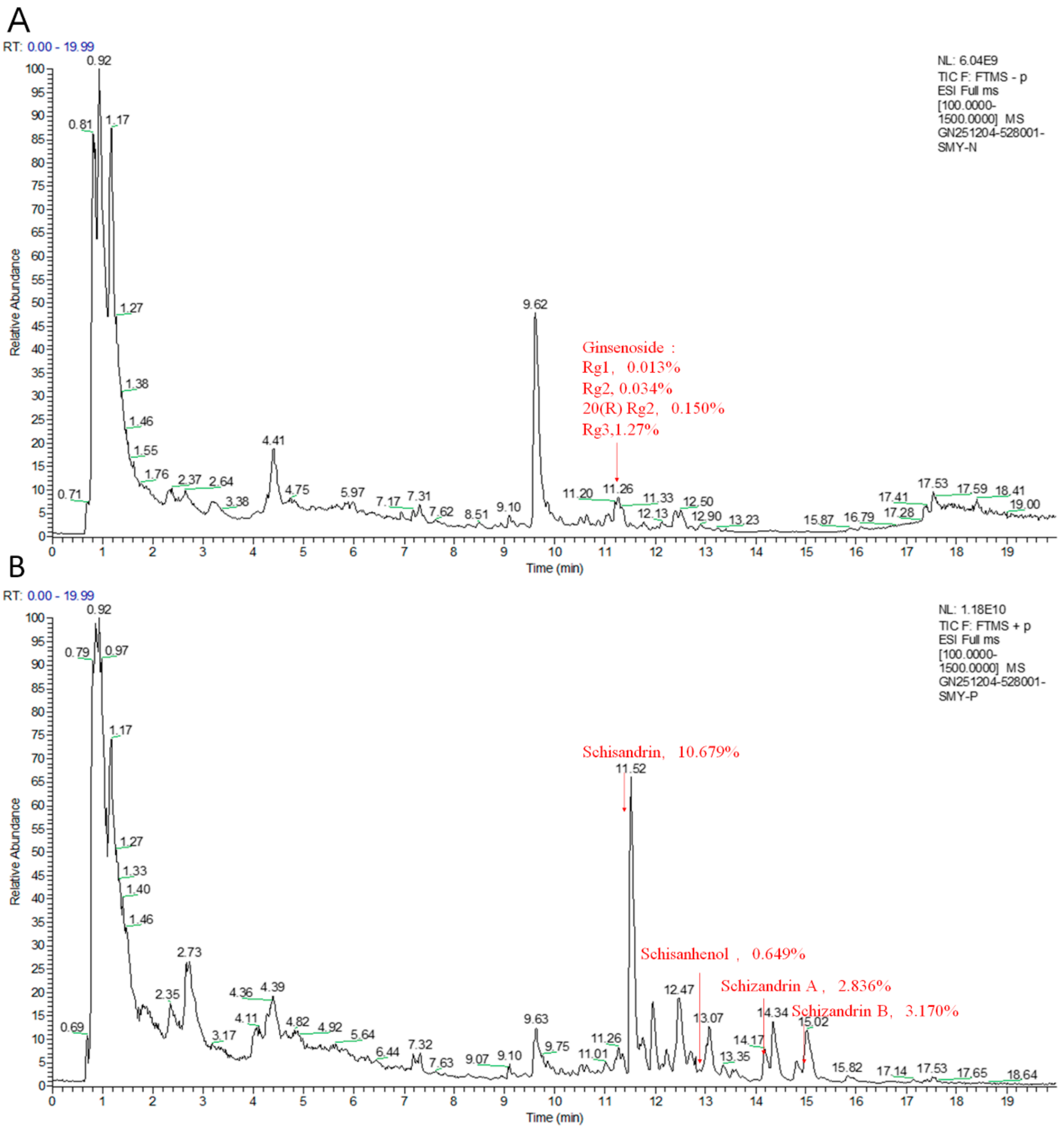


**Figure S2. UHPLC-MS/MS total ion chromatograms of SMY in negative-ion (A) and positive-ion (B) modes. Chromatographic separation was performed over 19.99 min using gradient elution, and electrospray-ionization mass spectra were acquired over m/z 100-1500. Major signals were tentatively assigned to characteristic lignans and ginsenosides; definitive compound identification and quantification require comparison with authenticated standards and full analytical validation.**

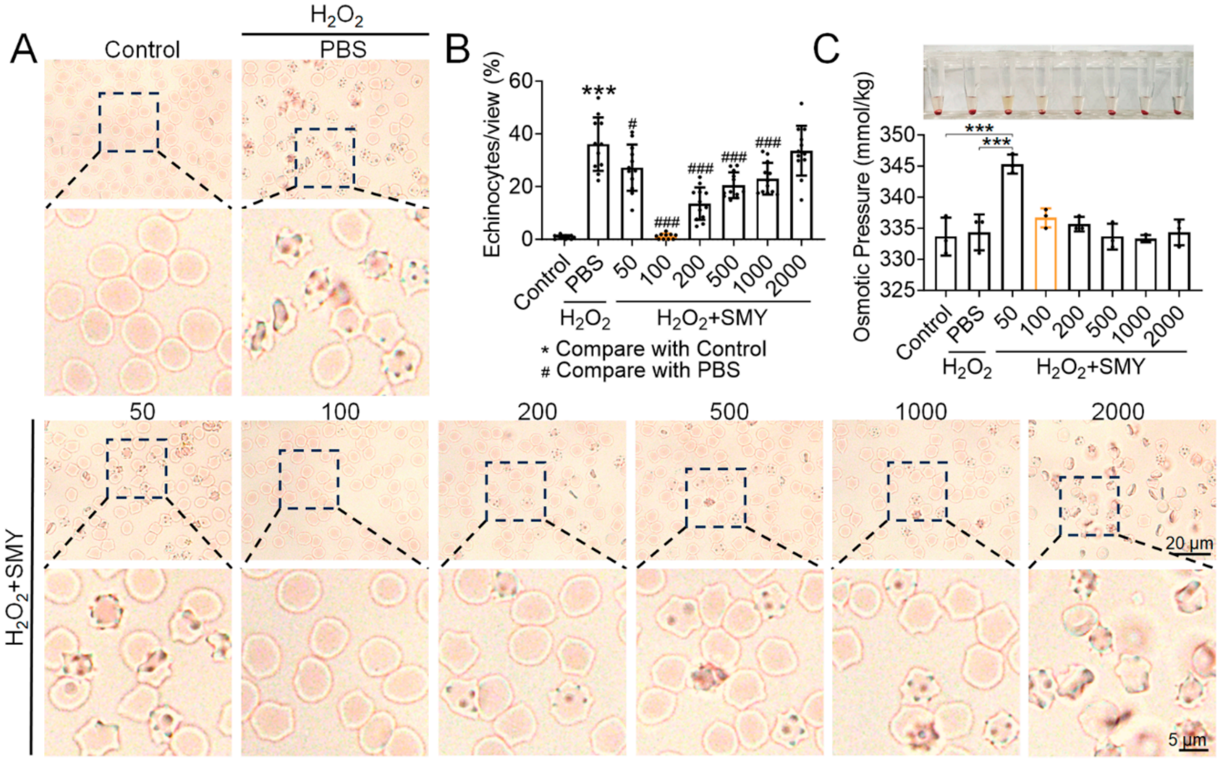


**Figure S3. SMY prevents $H_2O_2$-induced echinocytosis. (A) Representative erythrocyte morphology after $H_2O_2$ exposure with the indicated SMY dilutions. (B) Echinocyte percentages. (C) Osmolality of $H_2O_2$ solutions containing the indicated SMY dilutions. Data are presented as mean ± SD. *p < 0.05 and ***p < 0.001 versus control; ###p < 0.001 for the indicated comparison.**

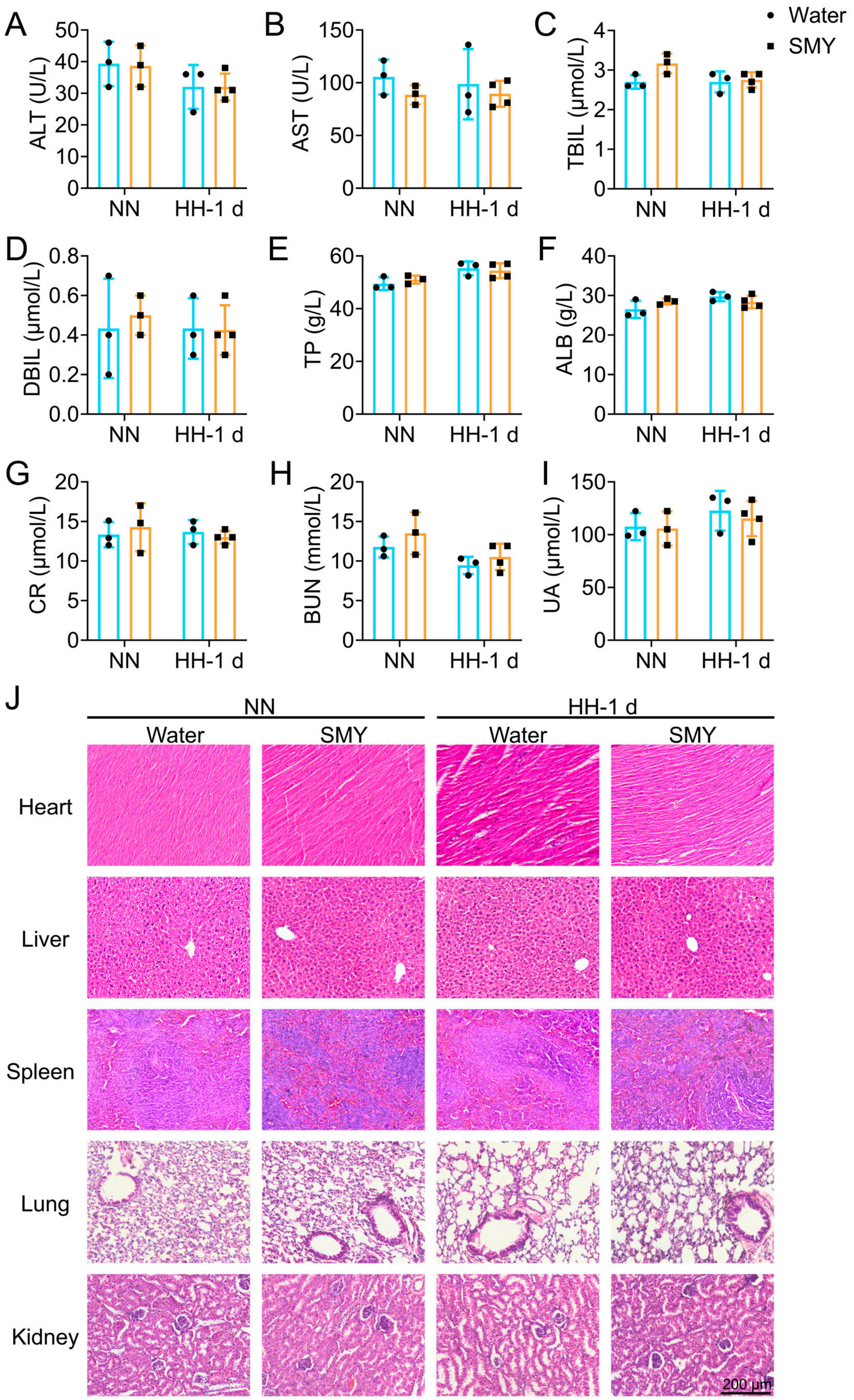


**Figure S4. Short-term SMY administration produces no detectable hepatic, renal, or histological toxicity. Mice received water or a 1:30 SMY preparation for 3 days before 24 h of HH. (A-F) Serum ALT, AST, TBIL, DBIL, TP, and ALB. (G-I) Serum creatinine, BUN, and UA. (J) Representative hematoxylin and eosin staining of heart, liver, spleen, lung, and kidney. Data are presented as mean ± SD. n = 3-6.**

**Table S1. Erythrocyte glutathione redox status following in vitro treatment of 1% $O_2$**

| Groups / Indexes | Nor-3h (n=4) | Hyp-3h (n=4) | Nor-6h (n=3) | Hyp-6h (n=3) | Nor-12h (n=3) | Hyp-12h (n=3) |
|---|---|---|---|---|---|---|
| **GSH（umol/L）** | 111.26±3.00 | 135.67±4.62 | 80.27±0.91 | 57.10±3.38 | 226.26±6.92 | 196.05±1.23 |
| **GSSG（umol/L）** | 0.64±0.04 | 0.55±0.05 | 0.79±0.02 | 0.49±0.04 | 0.98±0.04 | 0.75±0.02 |
| **T-GSH（umol/L）** | 112.54±3.03 | 136.76±4.69 | 83.85±1.23 | 58.08±2.50 | 228.22± 6.87 | 197.54±1.26 |
| **GSSG/GSH (%)** | 0.57±0.04 | 0.40±0.03 | 0.96±0.96 | 0.86±0.04 | 0.44±0.03 | 0.38±0.01 |
| **E_h'（mV）** | -189.47±0.95 | -196.59±0.70 | -178.95±0.12 | -175.67±0.23 | -202.06±1.15 | -201.94±0.11 |

$E_h'$ was calculated according to Colombo et al. (2010) using the Nernst equation: $E_h' = E°' + (RT/2F) \times \ln([GSSG]/[GSH]^2)$, with $E°' = -240$ mV at pH 7.4 and 25 °C. R is the gas constant, T is the absolute temperature, and F is the Faraday constant. Concentrations were converted to mol/L before calculation.
